# LC-MS and Spectrophotometric Approaches for Evaluation of Bioactive Compounds from Peru Cocoa By-Products for Commercial Applications

**DOI:** 10.3390/molecules25143177

**Published:** 2020-07-11

**Authors:** María de la Luz Cádiz-Gurrea, Álvaro Fernández-Ochoa, Francisco Javier Leyva-Jiménez, Noelia Guerrero-Muñoz, María del Carmen Villegas-Aguilar, Sandra Pimentel-Moral, Fernando Ramos-Escudero, Antonio Segura-Carretero

**Affiliations:** 1 Department of Analytical Chemistry, Faculty of Sciences, University of Granada, Av. Fuentenueva s/n, E-18071 Granada, Spain; alvaroferochoa@ugr.es (Á.F.-O.); nogumu_95@hotmail.es (N.G.-M.); marivillegas@ugr.es (M.d.C.V.-A.); spimentel@ugr.es (S.P.-M.); ansegura@ugr.es (A.S.-C.); 2 Functional Food Research and Development Center, Health Science Technological Park, Av. del Conocimiento s/n, E-18016 Granada, Spain; 3 Unidad de Investigación en Nutrición, Salud, Alimentos Funcionales y Nutraceúticos, Universidad San Ignacio de Loyola (UNUSAN-USIL), Calle Toulon 310, Lima 15024, Peru; diomedes.fernando@gmail.com; 4 Facultad de Ciencias de la Salud, Universidad San Ignacio de Loyola, Av. La Fontana 750, Lima 15024, Peru

**Keywords:** cocoa, polyphenols, LC-MS, by-products, food waste, revalorization, antioxidant

## Abstract

Peru is one of the main areas where there are large cocoa crops with special relevance to the economy of this country. In fact, cocoa is a major, economically important, international crop which has been linked to several benefits, such as anti-allergenic, anti-atherogenic, anti-inflammatory, anti-microbial, anti-oxidant, anti-thrombotic, cardioprotective and vasodilatory properties, relating to its bioactive compound content. However, in cocoa industrial processing, several residues or wastes, which are commonly discarded generating a negative impact on the environment, are produced in large amounts. Some of the cocoa by-products, which go underutilized, could be a good source of bioactive compounds with high utility for the development of innovative products in nutraceutical, medical or pharmaceutical industries. For this reason, the aim of this study is to qualitatively determine the phytochemical composition of husk and bean extracts from different cocoa-growing areas and processes from Peru by high performance liquid chromatography coupled to mass spectrometry. Furthermore, we aim to evaluate their phenolic and flavan-3-ol contents and antioxidant capacities for the purpose of highlighting the potential of cocoa by-products from these cultivars as functional ingredients. In total, 49 chemical compounds were detected in the analyzed extracts. Comparing both husks and beans, bean extracts were characterized by high content in flavonoids whereas husk extracts had a higher content of phenolic acids. The presence of these compounds together with the bioactivity results suggest that these matrices may be further studied for their revaluation in the development of high added-value products in nutraceutical, medical, and pharmaceutical industries.

## 1. Introduction 

The cocoa (*Theobroma cacao*) fruit is made up of a pod that includes around 40 seeds inside. These seeds are formed by the cocoa beans, which correspond to the cotyledon (internal part), and the cocoa husk, which is a thin layer that surrounds the bean [1]. After fermentation, roasting and grinding processes, the seeds give rise to different types of food that are frequently consumed, such as cocoa powders, chocolate and other cocoa-related products [1].

The high consumption of food derived from the cocoa fruit is due to its taste and aroma, but also to the large number of health benefits that have been demonstrated, such as cardioprotective [2], anti-inflammatory [3], anticancer [4], antiviral [5], antibacterial [6] and anti-neurodegenerative [7] properties, among others. These properties are largely linked to the antioxidant properties attributed to the bioactive phytochemicals contained in the cocoa fruit [3]. In cocoa beans, phenolic compounds represent about 10% of total constituents [8] and they are mainly made up of proanthocyanidins (58%), flavonols or flavan-3-ols (37%) and anthocyanins (4%) [9]. These secondary metabolites are of great interest since they possess high antioxidant activity with applicability in the food industry. Moreover, other biological properties have been ascribed to phenolic compounds from cocoa such as cardioprotective, immune-modulatory, anti-tumor and anti-inflammatory effects. In addition, there is clinical evidence on its beneficial effects on gut microbiota and the improvement of cognitive function in elderly people [10]. Most of these bioactive compounds are present in the bean, however, and others are generated or even modified during cocoa pre-treatments such as fermentation or roasting. Besides that, some phenolic compounds present in the cotyledons can migrate to the cocoa bean husk, producing an enriched by-product in these bioactive phytochemicals [11].

Nowadays, food industries produce massive amount of agri-food wastes that have a negative impact on the environment. Considering these effects along with the growing world population and the disappearance of raw materials, there could be a real threat of the reduction of food sources. Recently, there is increasing interest in these residues for their revaluation as food additives or supplements with a high nutritional added value [12,13]. In fact, the potentially marketable components in food wastes and by-products are a very important point for the majority of industries worldwide which are currently involved in the development of environmentally friendly protocols in order to reduce waste production [14]. In addition, research to enhance the exploitation of bioactive compounds from these wastes needs to be carried out for the development of high added-value products with low production costs and low environmental risks with potential application in different industries [15]. For these reasons, food manufacturers are developing several alternatives to give other uses to agri-food waste. These trends are commonly known as a circular bio-economy that is focused on using food waste for increasing the functional value of different products such as food, medicines, or cosmetics. Thereby, not only the products derived from cocoa are rich in phenolic compounds, but the by-products of its industry also contain a large amount of these phytochemicals, especially cocoa bean husks, which have also been related to a large number of bioactive effects [13]. However, the amount and proportions of each type of phenolic compound depend on the variety, the climate of the growing area and the type of processing that the cocoa fruit undergoes until the final cocoa product. For example, fermentation and drying of cocoa can be carried out under different conditions, affecting the bioactivity of the final cocoa powder [16,17,18]. In fact, when dry cocoa beans are obtained, several by-products are produced, which are mainly composed of the following three fractions: (i) cocoa pod husks, (ii) cocoa bean husks and (iii) cocoa mucilage. Among them, cocoa bean husks, which are the main by-product in the chocolate production, are generated after separation from the cotyledons during or after the pre-roasting process [13]. Some countries have legislation that regulates the maximum amount of this by-product that can be present in the cocoa mass. Taking into account the data reported by Rojo-Poveda et al. (2020), more than 700 thousand tons of cocoa bean husks are generated worldwide [19]. In most cases, these by-products are considered residues and generally left to dry on the plantations, which can lead to environmental problems, bad odors and even problems related to plant diseases like black pod rot [1]. A solution to these problems could be to implement revalorization approaches for cocoa by-products. For instance, in the case of cocoa bean husks, the main applications include the development of human dietary supplements, food additives, infusions, polyurethane foams for horticultural use, a source of pectins (to take advantage of its gelling and stabilizing properties) and animal feed [13,20]. 

Moreover, the new commitment to the revaluation of cocoa by-products may become important for the economy in countries with large cocoa plantations, such as Peru. In this country, the cocoa industry has a significant social relevance since cocoa is the sixth most important crop nationwide in terms of production and area harvested, according to ENA data for 2015 and 2016. However, a limited information on the phenolic profile and bioactivity of husk and bean cocoa extracts has been reported in Peru cultivars. Therefore, the main aim of this study is to qualitatively determine the phenolic composition of cocoa husk and bean extracts from different locations and fermentation processes by high-performance liquid chromatography (HPLC) coupled to high-resolution mass spectrometry (MS), and evaluate the antioxidant capacity for giving value to cocoa by-products from these regions in order to develop high-quality alternatives for opening new marketing channels. In this sense, this work will allow evaluating the antioxidant potential of cocoa by-products extracts as an abundant, inexpensive, renewable, and sustainable source using a non-toxic, accessible, and cheap methods for potential commercial applications in the development of new high added-value products in nutraceutical, medical and pharmaceutical industries.

## 2. Results and Discussion

### 2.1. Characterization of Cocoa Husk and Bean Extracts by HPLC-ESI-TOF

**Figure 1:**
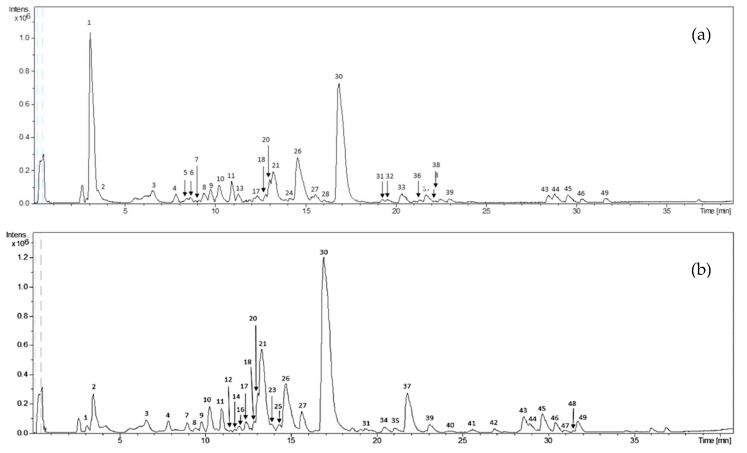
Base peak chromatograms of representative husk (**a**) and bean (**b**) extracts obtained by high-performance liquid chromatography coupled to electrospray ionization and time-of-flight mass spectrometry (HPLC-ESI-TOF-MS).

A comprehensive characterization of polar compounds of cocoa husk and bean extracts was carried out using advanced and powerful techniques. Figure 1 shows the representative base peak chromatograms (BPC) of cocoa husk (a) and bean (b) extracts. In addition, BPCs for all analyzed husk and bean extracts are shown in Figures S1 and S2.

All compounds were characterized by the interpretation of their mass spectra together with the information provided by the literature. Table 1 details information on all detected compounds numbered according to their elution order, including their retention times (RT), experimental *m*/*z*, molecular formulas, errors, σ values and if they were detected in each analyzed sample. The area under the curve of these compounds was integrated to estimate relatively their concentrations. These results are reflected in Table S1.

In the present study, a total of 49 compounds were detected and classified into the following four groups: phenolic acid derivatives, flavonoids, amino acid derivatives and other polar compounds.

#### 2.1.1. Phenolic Acid Derivatives

The high-resolution mass-spectrometry method used in this study enabled the characterization of a total of five phenolic acids and derivatives. In this scenario, four hydroxybenzoic acid derivatives have been detected at *m*/*z* 153 (peak **15**), 181 (peak **19**) and 329 (peaks **22** and **24**). These compounds were identified as protocatechuic acid, homovanillic acid and vanillic acid glycoside isomers, respectively. In addition, peak **28** was characterized as aesculetin, a lactone that derives from a cinnamic acid derivative. This compound as well as the previous mentioned ones have been previously found in *Theobroma cacao* extracts [21].

#### 2.1.2. Flavonoids

Flavonoids are the most relevant and complex group in cocoa, mainly flavan-3-ol subclass and proanthocyanidins. Among monomeric forms, catechin (peak **30**) and (epi)gallocatechin (peak **26**) were found at *m*/*z* 289 and 305, respectively. Polymeric and oligomeric forms of flavan-3-ol units linked by interflavan bonds play an important role in the formation of color, solubility and astringency of cacao [22]. Depending on this linkage, these compounds can be classified into A- or B- types. In this sense, peaks **40** and **48** were identified as isomers of procyanidin A-type with *m*/*z* at 575. 

Regarding B-type, procyanidins with different polymerization degrees were detected. Among these, three dimeric forms could be identified as procyanidin dimer isomers (peaks **21**, **25** and **37**) at *m*/*z* 577. In addition, five dimer derivatives were also characterized as (epi)catechin dimer hexose (peak **34**), arabinopyranosyl-(epi)catechin-(epi)catechin (peak **35**) and (epi)catechin methyl dimer isomers (peaks **39**, **46** and **49**). Trimer and tetramer procyanidins were detected with *m*/*z* 865 (peak **14**) and [M − 2H]^2−^ ions at *m*/*z* 576 (peaks **16** and **41**).

#### 2.1.3. Amino Acid Derivatives

Amino acid derivatives are generally found in cacao samples [23], and specifically seven of these compounds were identified in our study. Peaks **4**, **5** and **10** were characterized as tyrosine, fructose-leucine and phenylalanine, respectively.

On other hand, the occurrence of *N*-phenylpropenoyl-l-amino acids has been related to the astringent taste of cocoa. In addition, these compounds have demonstrated interesting biological properties such as being antioxidants or antimicrobials [24]. Peaks **13**, **20**, **27**, **31** and **42** were identified as *N*-caffeoyl-l-aspartate isomer 1 and 2, l-aspartic acid *N*-[3-(4-hydroxyphenyl)-1-oxo-2-propenyl], trans-clovamide (*N*-[(2*E*)-3-(3.4-dihydroxyphenyl)-1-oxo-2-propen-1-yl] and deoxyclovamide (*N*-[(2*E*)-3-(3.4-dihydroxyphenyl)-1-oxo-2-propen-1-yl]-l-tyrosine), respectively.

#### 2.1.4. Other Compounds

Among this heterogeneous group, two organic acids as gluconic acid (peak **1**) and citric acid (peak 3), sugars as peaks **2**, **8**, **9** and **36** and phlorotannin (peak **18**) were detected. Additionally, other compounds were also identified in the samples such as paeonol with *m*/*z* 165 (peak **38**), which is generally extracted by hydroalcoholic solvents and possesses potential anti-inflammatory, analgesic, antipyretic and immunomodulatory effects [25], and hexenyl primeveroside with *m*/*z* 393, which has been previously found in teas [26]. One of the most important alkaloids in cacao is theobromine, which was detected at *m*/*z* 179 (peak **11**).

#### 2.1.5. Hierarchical Clustering Analysis

**Figure 2:**
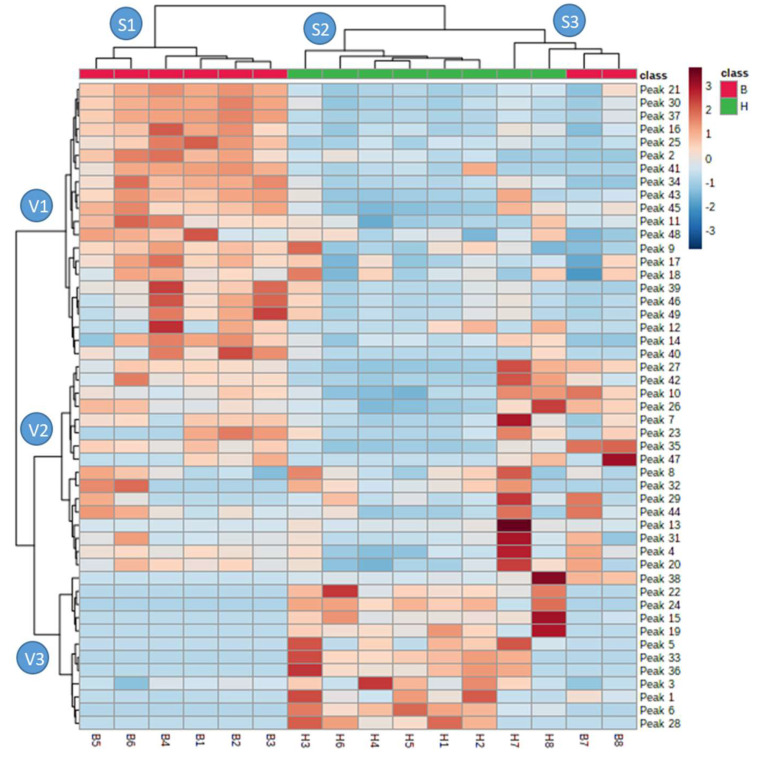
Hierarchical clustering analysis via heatmap using the areas under the curve of the 49 chemical compounds detected in the 16 cocoa by-product extracts.

Figure 2 shows the results of the hierarchical clustering analysis via heatmap. According to these results, three sample grouping clusters (S1, S2 and S3) have been obtained as well as three variable grouping clusters (V1, V2 and V3). Regarding the samples, most of the husk and bean cocoa extracts (samples 1–6) are grouped into two different clusters depending on the type of matrix (S1 and S2), respectively. The bean samples of the cluster S1 are characterized by presenting the highest concentrations of the variables grouped in cluster V1, which are mostly flavonoids. Regarding the husk samples, these are mostly characterized by the higher concentrations of the variables grouped in cluster V3, highlighting the presence of the five phenolic acids detected among them. In this sense, our results clearly revealed a higher flavonoid content in bean samples and a higher content of phenolic acids in husk samples. However, it is clearly shown that the samples 7 and 8 are grouped differently from the rest in the clustering analysis (cluster S3). These samples were those that presented lower results in the bioactivity tests (Table 2), and in general lower abundances in the detected phytochemical compounds (Table S1). This may be due to the fact that these samples are those obtained from regions of higher altitude within the samples with the genotype CCN51 from Leoncio Prado region (Perú). In fact, several studies have shown a negative correlation between the altitude and the synthesis of phytochemicals. For instance, Guerrero-Chavez et al. demonstrated this fact for the synthesis of anthocyanin from strawberry [27]. Nevertheless, these samples, especially sample H7, presented higher abundances of several compounds grouped in cluster V2 such as hydroxy-triaminoflavone, tri-*O*-methylsacarose, tyrosine, everlastoside C, hexenyl primeveroside or *N*-caffeoyl-l-aspartate isomers. The high presence of these compounds seems to be related to the particular conditions of this sample since these compounds presented lower amounts in the rest of the samples.

### 2.2. Total Phenol and Flavan-3-ol Contents and Antioxidant Capacities

As a previous step to measure the antioxidant capacity of husk and bean extracts, total phenol and flavan-3-ol contents were quantified by the Folin–Ciocalteau method and vanillin assay, respectively. It is worth mentioning that these methods are widely used as an approximate assay for semiquantitative phenolic compounds from plant extracts, although they have a weak accuracy [28].

The obtained values for each assay are shown in Table 2 and the results of the ANOVA tests are in Table S2. It is known that the variety and quantity of phenolic compounds in cocoa extracts depend not only on intrinsic factors such as genotype, but also extrinsic factors such as origin, climatic conditions of crops, harvest season, drying and fermentation methods and other processing steps [29,30]. Moreover, the complex chemical structure of phenolic compounds and their extraction from vegetal tissues are important factors to take into account. In this sense, low-molecular-weight phenols such as flavonoids, hydroxycinnamic or benzoic acids in free form or as glycosides are easily extractable, whereas tannins, such as proanthocyanidins, present low solubility and matrix availability [31]. As it is impossible to develop an extraction method suitable for all phenolic compounds from plant sources, extraction becomes a key step in the obtainment of antioxidant compounds, with hydroalcoholic solvents such as methanol/water generally used for this purpose [32]. As a result, the highest values of TPC and TFC were obtained in cocoa bean extracts, specifically B1, B2, B4 and B6, corresponding to La Convención, Zarumilla, and Leoncio Padro (Huayranga and Cadena crop locus of CCN51 genotypes), respectively. Both B4 and B6 samples were fermented in polypropylene bags for 5 days and dried by direct sunlight, whereas B1 and B2 were fermented in wooden boxes (6 days) and in polypropylene bags with a wooden platform (7 days), respectively, and both cocoa samples were sun-dried under a plastic film cover (Agro Films). Interestingly, cocoa husk extract H3 showed a higher TPC value than its correspondent bean extract (B3). On the other hand, B7 sample, from the same genotype CCN51, showed the lowest values in bean extracts. In fact, the majority of the values of husk extracts were higher than B7. This behavior was similar in the B8 sample, where both cacao beans were cultivated at more than 830 m.a.s.l. without a plastic film cover and in polypropylene bags instead of wooden boxes. Moreover, the effect of the cocoa fermentation days could also affect the results according to the data reported by Barrientos et al. 2019. This work showed a decrease in TPC after 137 h of fermentation and drying [33]. However, in our case, B1 (6 days) and B2 (7 days) obtained higher values than B7 or B8 with 5 and 4 days of fermentation, respectively.

On the other hand, ferric reducing antioxidant power (FRAP) and Trolox equivalent antioxidant capacity (TEAC) methods were used to determine the antioxidant capacity of the extracts by a single-electron transfer mechanism directly and indirectly, respectively. TEAC has become one of the most widely used assays in evaluating the antioxidant capacity of food bioactive compounds from different sources and FRAP is also one of the most preferred assays to test the antioxidant ability of several food components since this method is rapid, robust and inexpensive [34]. In general, the obtained results (Table 2) revealed a higher antioxidant activity of bean extracts compared to husk extracts. This trend can be explained based on the total phenolic or flavon-3-ols contents of the samples, as revealed by the Folin–Ciocalteau or vanillin assays, respectively. In contrast, the extraction yields were significantly higher in husk extracts than in bean extracts. Therefore, this showed that there was no direct relationship between the extraction yields of these matrices and their antioxidant capacity. Rather, the antioxidant capacity is more closely related to the specific chemical compounds present in the matrices or to the synergistic effects that can occur between them [35]. Although the comparison of our results with those reported in the literature could be untenable due to differences in the properties of the sample, applied technologies, extraction systems, and assay methodologies, previous reports have shown similar results. For example, Hernández-Hernández et al. 2019 tested the TPC of cocoa bean husks in several Mexican genotypes in 2014 and 2015 [36]. TPC for cocoa genotypes in 2014 ranged from 3.86 ± 0.54 to 17.34 ± 3.49 μg gallic acid /mg dry husk cocoa extract. These results were slightly lower than our values. However, in 2015, the values were a bit higher [36]. 

**Figure 3:**
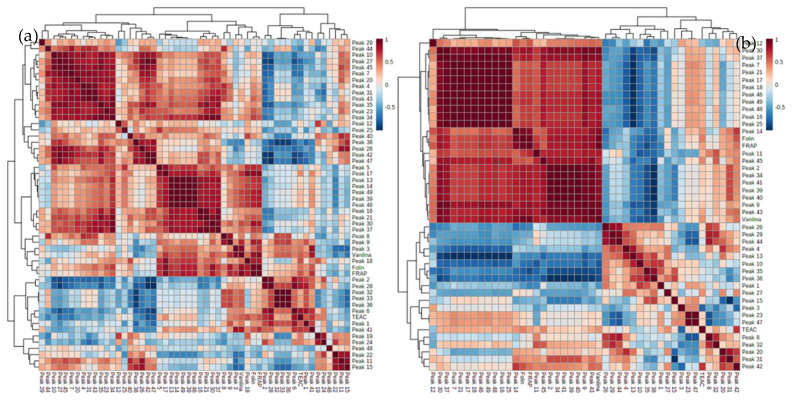
Correlation analysis using Pearson distance. (**a**) Husk and (**b**) bean samples. (Intense red or blue colors show positive or negative correlations, respectively).

In order to know which compounds are more closely related to the antioxidant activity, a correlation analysis was performed for each matrix using the relative concentrations of the compounds and the results of antioxidant activity. Figure 3 shows the correlation matrices obtained in these analyses for each kind of matrix. Observing both correlation matrices, several compounds stand out for having a high positive correlation with the results obtained in the FRAP assay. These compounds are as follows: catechin, procyanidin C, aspalatin, fructofuranosyl-treonyl-glucopyranoside, phlorotannin and several (epi)catechin and procyanidin derivates. In addition, we also performed a clustering analysis using the results of both matrices (Figure S3), which showed a high correlation of procyanidin derivates, catechin, theobromine, quercetin glucoside and quercetin arabinofuranoside with the data obtained from the spectrophotometric assays. 

The bioactivity of several of these compounds has been extensively studied previously [37]. For example, the bioactivity of the flavan-3-ols catechin, epigallocatechin and epicatechin on multiple targets has been demonstrated. Cornelia Braicu et al. studied the relationship between the structure of these flavan-3-ols and their biological activities, revealing a dose- and time-dependent inhibitory effect in a human breast cancer line (Hs578T) [38]. Another study showed the antibacterial activity of these catechin derivatives [39]. On the other hand, different pharmacological properties have been attributed to theobromine such as being an anticarcinogenic or antioxidant, as well as its beneficial effects against cardiovascular diseases [40]. The bioactivity of phlorotannin has also been explored, revealing excellent properties such as anti-inflammation, anti-cancer, anti-allergic or anti-wrinkling, among others, that have given it great potential to consider it a source for the development of cosmeceuticals [41]. The bioactive properties of procyanidin derivates isolated from *Uncaria tomentosa* have been also studied, revealing their cytotoxic properties in colon adenocarcinoma and gastric cancer cell lines [42].

The relationship of these compounds with the results of antioxidant capacity added to the fact that most of them are the majority compounds in the bean extract samples (e.g., catechin, epigallocatechin, procyanidin dimer type B, etc.) (Figure 2), means that bean extracts present significantly higher bioactivities than the husk samples. Therefore, it has been demonstrated that the cocoa bean samples present a high concentration of compounds with high bioactive potential, such as flavonoids, leading to better bioactivity results. On the other hand, H3 extract composition, which also gave high antioxidant capacity values, was highly represented by phenolic acids such as vanillic acid derivatives. It is known that the chemical structure of phenolic compounds and their radical-scavenging activities are directly related. In this sense, proanthocyanidins are potent antioxidants due to their extensive electron delocalization induced by the catechol unit on B-ring and *o*-hydroxy phenolic groups in their structures. This capacity improves with the polymerization degree [23]. However, the glycosylation of these compounds reduces their activity compared to aglycones, whereas substitutions of the 4-hydroxy group of homovanillic acid by a methoxy derivative enhance the antioxidant effectiveness of this compound compared with vanillic acid [43]. All these results suggest that these by-products are very valuable for their revaluation in the development of value-added products, such as functional foods, nutraceuticals, cosmeceuticals, etc.

As shown in the cluster S2 in the heatmap (Figure 2), husk extracts have been characterized by the high presence of the following compared to bean extracts: vanillic acid glycosides, protocatechuic acid, fructose-leucine, everlastoside C, β-d-Glucopyranoside, 2-phenylethyl 6-*O*-β-d-xylopyranose, aesculetin and homovanillic, citric and gluconic acids. Some of these compounds have also shown positive correlations with the biological activity variables such as vanillic acid glycosides, fructose-leucine, citric acid, aesculetin or gluconic acid, among others (Figure 3). Some recent studies have revealed the bioactive potential of some of these compounds. For instance, aesculetin has been proposed as one of the main substances responsible for the antioxidant properties of *Plantago asiatica L* [44]. In this study, in vitro antioxidant assays in cells were performed with the isolated aesculetin, demonstrating its ability to reduce damage in cocoa-2 cells produced by H_2_O_2_. It showed that this compound protected oxidative stress by activation of Nrf-2 and SOD, CAT y GCS genes. On the other hand, Ja Kim et al. studied the antioxidant capacity of a vanillic acid glycoside isolated from *Gardeniae jasminoides*, revealing a moderate antioxidant potential of this compound in DPPH and superoxide anion radical scavenging assay systems [45]. According to this evidence, the presence of these compounds in the cocoa husk extracts implies that this by-product can be reused to obtain value-added products due to their antioxidant capacity.

## 3. Materials and Methods

### 3.1. Chemicals

All reagents used in this work were analytical grade and used without changes. The analytical procedures were performed using water purified by a Milli-Q system from Millipore (Bedford, MA, USA). LC-MS grade methanol and acetic acid were purchased from Fisher Chemicals (Waltham, MA, USA) and Sigma-Aldrich (Steinheim, Germany), respectively.

For TPC and TFC and antioxidant capacity, the following reagents were provided from the indicated suppliers: gallic acid, sodium acetate, ferric chloride and hydrochloric acid were purchased from Panreac (Barcelona, Spain) and ABTS [2,2-azinobis-(3-ethylbenzothiazoline-6-sulphonate)], ferric sulphate, Folin–Ciocalteu reagent, potassium persulfate, TPTZ (2,4,6-tripyridyl-S-triazine), Trolox (6-hydroxy-2,5,7,8-tetramethylchroman-2-carboxylic acid), vanillin and (+)-catechin from Sigma-Aldrich (St. Louis, MO, USA).

### 3.2. Plant Collection and Extract Preparation

**Figure 4:**
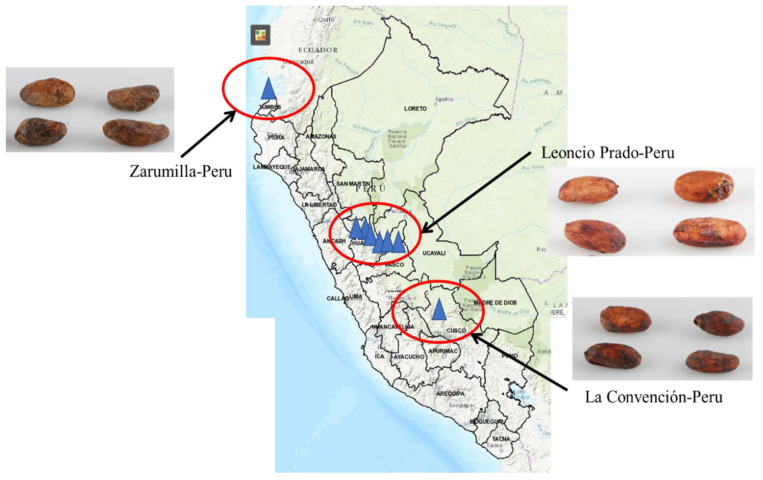
Different origins of cacao samples in Peru.

All cocoa samples were chosen based on geographical diversity in Peru, their production and availability. In addition, the origin (Figure 4), crop, drying types and methods of each genotype are described in Table 3. After pre-treatment process, husks were separated to the cocoa bean and each sample was ground using a laboratory mill. 

For extract preparation, 1 g of each sample (8 husks and 8 beans) was added into 10 mL of methanol:water (80:20, *v*:*v*). The samples were vortexed for 1 min, sonicated for 10 min, centrifuged for 10 min at 7700× *g* and, finally, the supernatants were collected. This process was repeated 3 times and the three supernatants of each sample were mixed, filtered and evaporated to dryness under vacuum in Speed Vac (Thermo Scientific® SC 250 exp). The residues were weighed and dissolved in the extraction solvent mixture at 5 mg/mL and stored at −20 °C until further use. Moreover, they were filtered through a 0.25 μm filter to analyze them by LC-MS.

### 3.3. HPLC-ESI-TOF-MS Analysis

The qualitative characterization of 16 different *T. cacao* extracts was carried out using a RRPC 1200 series (Agilent Technologies, Palo Alto, CA, USA) following the method reported by Cádiz-Gurrea et al. 2019 [46] with minor changes. Briefly, the multistep gradient used to separate the phytochemicals was as follows: 0 min, 0% B; 5 min, 25% B; 20 min, 39% B; 30 min, 60% B; 38 min, 100% B, 42 min, 0% B. Finally, a conditioning cycle (10 min) was applied with initial conditions before each injection. The injection volume was 10 µL and the separation was performed at room temperature. The total flow rate was fixed at 0.5 mL/min and therefore the use of a “T” type splitter was required for coupling with a time of flight mass spectrometer (microTOF, Bruker Daltonik, Bremen, Germany), which was equipped with a orthogonal electrospray interface (ESI) (model G1607 from Agilent Technologies, Palo Alto, CA, USA) operating in negative ionization mode. Measurements were made in triplicate.

### 3.4. Total Phenolic and Flavan-3-ol Content

Total phenolic and flavan-3-ol contents were determined by the Folin–Ciocalteu and vanillin methods, respectively, reported by Cádiz-Gurrea et al. (2017) [3]. The phenol content was calculated based on the calibration curves of gallic acid (GAE) and expressed as μg GAE/mg of dry extract, and flavan-3-ol content was calculated based on the calibration curves of (+)-catechin and expressed as μg CE/mg of dry extract. Measurements were made in triplicate.

### 3.5. Antioxidant Capacity Measurements

For the evaluation of the antioxidant capacity, different assays were carried out using previously described methods [3,23]. These methods were: TEAC assay, which measures the reduction of the radical cation of 2,2′-azinobis (3-ethylbenzothiazoline-6-sulphonate) (ABTS) by antioxidants using Trolox as the standard, and FRAP assay where antioxidant values were calculated using FeSO_4_·7H_2_O as the standard. Measurements were made in triplicate.

### 3.6. Data Processing and Statistics

All samples prepared in triplicate were aggregated and presented as mean ± standard deviation (SD). Chromatograms were processed in DataAnalysis Version 4.0 software (Bruker Daltonics, Bremen, Germany) in which the areas of the detected compounds were integrated and exported. Compounds were identified by comparing the MS data with that reported in the literature. IBM SPSS Statistics 23 software (Madison St. Chicago, IL, USA) was employed to perform one-way analysis of variance (ANOVA) at a 95% confidence level (*p* ≤ 0.05) in order to analyze statistically significant differences among the TPC, TFC and antioxidant activities of the extracts (Table S2). A hierarchical clustering analysis via heatmap was performed using compound variables to identify groupings between samples. This analysis was performed using a Pearson distance and a Ward clustering algorithm. In addition, the area under the curve obtained for each compound in each sample (Table S1), together with the results of the antioxidant tests, were grouped in a single file. All these data were subjected to a correlation test using a distance measure of Pearson. All variables were previously log-transformed and auto-scaled so that the variables were normally distributed. The hierarchical analysis and the correlation analysis were performed in Metaboanalyst 4.0 software [47].

## 4. Conclusions

Since the revalorization of cocoa by-products may become important for the economy in Peru, a country with large cocoa plantations, this study presents interesting results for the possible development of future valuable applications from husk and bean by-products. By the interpretation of mass spectrometry data and information available in the literature, a total of 49 compounds were characterized in the studied matrices, which were classified into four groups: phenolic acid derivatives, flavonoids, amino acid derivatives, and other polar compounds. In general, the bean samples showed a high flavonoid content and, on the other hand, the husk samples presented a higher richness of phenolic acids. Therefore, bean by-products have a predictably greater potential for the development of new high added-value products such as nutraceuticals, functional foods, cosmeceuticals, etc.

Most of the samples analyzed showed similar behaviors except those that were grown in high altitude regions or dried by direct sunlight and fermented in polypropylene bags, which presented lower flavonoid contents and antioxidant activities. Moreover, the results obtained by TEAC and FRAP assays have shown a high correlation with several of the compounds analyzed, such as catechin, epicatechin, theobromine, etc., demonstrating the possible high bioactive potential of these compounds. In general, higher bioactivity values were obtained in samples from Leoncio Padro origin (Genotype CCN51), which is an interesting result for the future selection of by-products in the event that they are to be exploited for the development of high added-value applications. 

These by-products could be applied in the food industry after toxicity studies that guarantee their food safety as an ingredient rich in phenolic compounds with high antioxidant activity, as a natural colorant and flavoring agent, or even also as a functional ingredient with high value in cosmetic and pharmaceutical industries.

## Figures and Tables

**Table 1:** Chemical characterization of compounds detected in husk and bean cocoa extracts by HPLC-ESI-TOF-MS.

Peak	RT (min)	*m*/*z* exp	*m*/*z* cal	Error (ppm)	mSigma	Molecular Formula	Proposed Compound	H1	H2	H3	H4	H5	H6	H7	H8	B1	B2	B3	B4	B5	B6	B7	B8
**1**	3.1	195.0516	195.0510	2.9	2.6	C_6_ H_12_ O_7_	Gluconic acid	X	X	X	X	X	X	X	X	X	X	X		X	X	X	X
**2**	3.5	341.1086	341.1089	0.8	29.5	C_12_ H_22_ O_11_	Sacarose	X	X	X	X	X	X	X	X	X	X	X	X	X	X		
**3**	6.6	191.0198	191.0197	0.1	0.6	C_6_ H_8_ O_7_	Citric acid	X	X	X	X	X	X	X	X	X	X	X	X	X		X	X
**4**	7.9	180.0670	180.0666	2.1	0.6	C_9_ H_11_ NO_3_	Tyrosine	X	X	X	X	X	X	X	X	X	X	X	X	X	X	X	X
**5**	8.4	292.1405	292.1402	0.9	15.4	C_12_ H_23_ NO_7_	Fructose-leucine	X	X	X	X			X	X								
**6**	8.6	265.0935	265.0929	2.2	2.0	C_10_ H_18_ O_8_	Unknown 1	X	X	X	X	X	X	X	X								
**7**	9.0	282.0872	282.0884	4.2	25.7	C_15_ H_13_ N_3_ O_3_	Hydroxy-triaminoflavone	X	X	X	X	X	X	X	X	X	X	X	X	X	X		X
**8**	9.5	383.1559	383.1559	0.0	11.1	C_15_ H_28_ O_11_	Tri-*O*-methylsacarose	X	X	X	X	X	X	X	X	X	X	X	X	X	X	X	X
**9**	9.8	442.1566	442.1566	0.0	0.0	C_16_ H_29_ NO_13_	Fructofuranosyl-treonyl-glucopyranoside	X	X	X	X	X	X	X	X	X	X	X	X	X	X	X	X
**10**	10.2	164.0718	164.0717	0.6	2.3	C_9_ H_11_ NO_2_	Phenylalanine	X	X	X	X	X	X	X	X	X	X	X	X	X	X	X	X
**11**	10.9	179.0580	179.0574	3.1	2.2	C_7_ H_8_ N_4_ O_2_	Theobromine	X	X	X	X	X	X	X	X	X	X	X	X	X	X	X	X
**12**	11.3	451.1245	451.1246	0.1	5.6	C_21_ H_24_ O_11_	Aspalathin isomer 1	X	X						X		X	X	X				
**13**	11.3	294.0626	294.0619	2.2	6.9	C_13_ H_13_ NO_7_	*N*-caffeoyl-l-aspartate isomer 1	X	X	X	X	X		X	X							X	
**14**	11.7	865.1971	865.1985	1.6	23.1	C_45_ H_38_ O_18_	Procyanidin C isomer 1	X	X	X	X	X		X	X	X	X	X	X		X		
**15**	11.7	153.0202	153.0193	5.7	1.1	C_7_ H_6_ O_4_	Protocatechuic acid	X	X	X	X	X	X	X	X								X
**16**	12.0	576.1274	576.1273	0.3	159.7	C_60_ H_50_ O_24_	(Epi)catechin tetramer isomer 1	X	X	X	X	X	X	X	X	X	X	X	X	X	X		X
**17**	12.4	451.1254	451.1246	1.7	3.1	C_21_ H_24_ O_11_	Aspalathin isomer 2	X	X	X	X	X	X	X	X	X	X	X	X	X	X		X
**18**	12.8	369.028	369.0252	7.6	18.9	C_18_ H_10_ O_9_	Phlorotannin	X	X	X	X	X	X	X	X	X	X	X	X	X	X		X
**19**	12.9	181.0514	181.0506	4.2	9.8	C_9_ H_10_ O_4_	Homovanillic acid	X	X	X	X	X	X	X	X								
**20**	13.0	294.0622	294.0619	0.8	3.1	C_13_ H_13_ NO_7_	*N*-caffeoyl-l-aspartate isomer 2	X	X	X	X	X	X	X	X	X	X	X	X	X	X	X	X
**21**	13.3	577.1353	577.1351	0.2	1.7	C_30_ H_26_ O_12_	Procyanidin dimer type B isomer 1	X	X	X	X	X	X	X	X	X	X	X	X	X	X		X
**22**	13.4	329.0893	329.0878	4.6	40.8	C_14_ H_18_ O_9_	Vanillic acid glycoside isomer 1	X	X	X	X	X	X	X	X								
**23**	13.9	451.1233	451.1246	2.9	9.0	C_21_ H_24_ O_11_	Aspalathin isomer 3		X	X				X	X	X	X	X					X
**24**	14.1	329.0896	329.0878	5.5	30.0	C_14_ H_18_ O_9_	Vanillic acid glycoside isomer 2	X	X	X	X	X	X	X	X								
**25**	14.3	577.1343	577.1351	1.4	34.5	C_30_ H_26_ O_12_	Procyanidin dimer type B isomer 2	X	X		X			X	X	X	X	X	X	X	X		X
**26**	14.6	305.0675	305.0667	2.7	26.3	C_15_ H_14_ O_7_	(Epi)gallocatechin	X	X	X	X	X	X	X	X	X	X	X	X	X	X	X	X
**27**	15.6	278.0668	278.0670	0.9	1.7	C_13_ H_13_ NO_6_	l-aspartic acid *N*-[3-(4-hydroxyphenyl)-1-oxo-2-propenyl]	X	X	X	X	X	X	X	X	X	X	X	X	X	X	X	X
**28**	16.0	177.0200	177.0193	3.8	3.6	C_9_ H_6_ O_4_	Aesculetin	X	X	X	X	X	X										
**29**	16.3	407.1552	407.1559	1.7	30.5	C_17_ H_28_ O_11_	Unknown 2		X	X	X	X	X	X	X					X	X	X	
**30**	16.8	289.0726	289.0718	2.8	5.8	C_15_ H_14_ O_6_	Catechin	X	X	X	X	X	X	X	X	X	X	X	X	X	X	X	X
**31**	19.3	358.0927	358.0932	1.5	4.8	C_18_ H_17_ NO_7_	Trans-clovamide (*N*-[(2*E*)-3-(3.4-dihydroxyphenyl)-1-oxo-2-propen-1-yl] -3-hydroxy-l-tyrosine)	X	X	X	X	X	X	X	X	X	X	X	X	X	X	X	
**32**	19.5	381.1760	381.1766	1.7	1.8	C_16_ H_30_ O_10_	Everlastoside C isomer 1	X	X	X	X	X	X	X						X	X		
**33**	20.3	381.1760	381.1766	1.2	6.4	C_16_ H_30_ O_10_	Everlastoside C isomer 2	X	X	X	X	X	X	X									
**34**	20.4	737.1735	737.1723	1.6	10.3	C_36_ H_34_ O_17_	(Epi)catechin dimer hexose	X	X	X	X	X	X	X	X	X	X	X	X	X	X		
**35**	21.0	707.1641	707.1618	3.4	4.1	C_35_ H_32_ O_16_	Arabinopyranosyl-(epi)catechin-(epi)catechin	X	X	X	X	X	X	X	X	X	X	X	X	X	X	X	X
**36**	21.3	415.1589	415.1610	5.0	31.9	C_19_ H_28_ O_10_	β-d-Glucopyranoside, 2-phenylethyl 6-*O*-β-d-xylopyranose	X	X	X	X	X	X	X									
**37**	21.6	577.1339	577.1351	2.2	4.1	C_30_ H_26_ O_12_	Procyanidin dimer type B isomer 3	X	X	X	X	X	X	X	X	X	X	X	X	X	X	X	X
**38**	22.1	165.0560	165.0557	1.6	4.4	C_9_ H_10_ O_3_	Paeonol	X	X	X	X	X	X	X	X							X	X
**39**	23.1	605.1679	605.1664	2.4	4.7	C_32_ H_30_ O_12_	(Epi)catechin methyl dimer isomer 1	X	X	X	X	X		X	X	X	X	X	X	X	X		
**40**	24.0	575.1181	575.1195	2.3	126.2	C_30_ H_24_ O_12_	Procyanidin dimer type A isomer 1			X					X	X	X	X	X	X	X		
**41**	25.5	576.1296	576.1273	3.9	29.1	C_60_ H_50_ O_24_	(Epi)catechin tetramer isomer 2		X	X	X	X				X	X	X	X	X	X		
**42**	26.8	326.1039	326.1034	1.4	12.1	C_18_ H_17_ NO_5_	Deoxyclovamide (*N*-[(2*E*)-3-(3.4-dihydroxyphenyl)-1-oxo-2-propen-1-yl]-l-tyrosine)	X	X	X	X	X	X	X	X	X	X	X	X	X	X	X	X
**43**	28.4	463.0878	463.0882	0.9	3.2	C_21_ H_20_ O_12_	Quercetin glucoside	X	X	X	X	X	X	X	X	X	X	X	X	X	X	X	X
**44**	28.8	393.1778	393.1766	3.1	9.5	C_17_ H_30_ O_10_	Hexenyl primeveroside	X	X	X	X	X	X	X	X	X	X	X	X	X	X	X	X
**45**	29.5	433.0774	433.0776	0.5	12.4	C_20_ H_18_ O_11_	Quercetin arabinofuranoside	X	X	X	X	X	X	X	X	X	X	X	X	X	X	X	X
**46**	30.3	605.1643	605.1664	3.6	7.8	C_32_ H_30_ O_12_	(Epi)catechin methyl dimer isomer 3	X	X	X	X	X		X	X	X	X	X	X	X	X		X
**47**	30.9	516.2475	516.2450	4.8	13.4	C_24_ H_39_ NO_11_	Unknown 3							X	X	X	X	X					X
**48**	31.3	575.1190	575.1195	1.0	35.6	C_30_ H_24_ O_12_	Procyanidin dimer type A isomer 2	X		X	X	X	X	X	X	X	X	X	X	X	X		X
**49**	31.6	605.1664	605.1671	1.1	10.3	C_32_ H_30_ O_12_	(Epi)catechin methyl dimer isomer 4	X	X	X	X	X		X	X	X	X	X	X	X	X		X

RT, retention time; X, occurrence; H, cocoa husks extracts; B, cocoa bean extracts.

**Table 2:** Extraction yields and spectrophotometric results of cocoa extracts. Value = mean value ± SD.

Sample	Yield (%)	Folin–Ciocalteau (µg GAE/mg Dry Extract)	Vanillin (µg CE/mg Dry Extract)	FRAP (µg eq. FeSO_4_/mg Dry Extract)	TEAC (µg eq. Trolox/mg Dry Extract)
H1	19.3	9.3 ± 0.8	22 ± 2	0.073 ± 0.003	0.9 ± 0.1
H2	20.9	14.8 ± 0.7	32 ± 9	0.11 ± 0.01	0.96 ± 0.02
H3	17.9	22.2 ± 0.7	36 ± 2	0.131 ± 0.003	1.048 ± 0.001
H4	23.3	11.4 ± 0.2	33 ± 1	0.096 ± 0.006	1.1 ± 0.1
H5	16.0	7.8 ± 0.4	24.9 ± 0.5	0.069 ± 0.003	0.618 ± 0.005
H6	17.4	4.9 ± 0.2	16.1 ± 0.8	0.044 ± 0.004	0.5 ± 0.1
H7	8.6	10.7 ± 0.1	27 ± 6	0.078 ± 0.003	0.3 ± 0.1
H8	7.9	8.6 ± 0.1	16 ± 1	0.065 ± 0.008	0.44 ± 0.02
B1	10.6	29.8 ± 0.7	96 ± 8	0.195 ± 0.005	1.54 ± 0.04
B2	10.3	28.1 ± 0.4	99 ± 2	0.23 ± 0.01	1.4 ± 0.3
B3	7.9	20.9 ± 0.1	74 ± 8	0.157 ± 0.008	1.1 ± 0.4
B4	11.0	31.3 ± 0.1	130 ± 2	0.266 ± 0.008	1.0 ± 0.1
B5	8.7	15.0 ± 0.1	50.6 ± 0.5	0.128 ± 0.005	1.03 ± 0.01
B6	10.0	29.5 ± 0.5	118 ± 16	0.216 ± 0.002	2.2 ± 0.1
B7	15.8	11.4 ± 0.1	19 ± 2	0.095 ± 0.003	1.07 ± 0.02
B8	8.0	13.7 ± 0.2	34.6 ± 0.2	0.116 ± 0.001	1.4 ± 0.1

H: husk cocoa extracts; B: bean cocoa extracts. FRAP: ferric reducing antioxidant power assay; TEAC: Trolox equivalent antioxidant capacity; GAE: gallic acid equivalents; CE: catechin equivalents; eq.: equivalents.

**Table 3:** Origin, crop, drying types and methods of each genotype of cocoa samples.

Origin	La Convención	Zarumilla	Leoncio Prado					
Genotype	CHUNCHO	TRINITARIO	CCN51					
N° samples	1	2	3	4	5	6	7	8
Crop locus	Quillabamba	Papayal	Km. 51	Huayranga	Pumahuasi	Cadena	La Victoria	Las Vegas
Altitude (m.a.s.l.)	950	42	710	670	720	700	830	900
Type of fermentation & days	Wooden boxes (6 days)	Polypropylene bags with wooden platform (7 days)	Polypropylene bags (5 days)	Polypropylene bags (5 days)	Polypropylene bags (4 days)	Polypropylene bags (5 days)	Polypropylene bags (5 days)	Polypropylene bags (4 days)
Drying methods	Sun-dried with plastic film cover (Agro Films)	Sun-dried with plastic film cover (Agro Films)	Drying by direct sunlight	Drying by direct sunlight	Drying by direct sunlight	Drying by direct sunlight	Drying by direct sunlight	Drying by direct sunlight

## Supplementary Materials

The following are available online, Figure S1: Base peak chromatogram of husk extracts, Figure S2: Base peak chromatogram of bean extracts, Figure S3: Correlation analysis using Pearson distance using all data obtained from husk and bean samples. (Intense red or blue colors show positive or negative correlations, respectively), Table S1: Abundance of compounds from *Theobroma cacao* extracts by HPLC-ESI-TOF, Table S2. ANOVA data for TPC, TFC and antioxidant assays of husk and bean extracts.
