# Supplementary material for: LC-MS and Spectrophotometric Approaches for Evaluation of Bioactive Compounds from Peru Cocoa By-Products for Commercial Applications
Source: Molecules. 2020 Jul 11;25(14):3177. doi: 10.3390/molecules25143177 (PMC7397285; doi:10.3390/molecules25143177)
Supplement: Supplementary file 1 [file molecules-25-03177-s001.pdf]

molecules

Article

LC-MS and spectrophotometric approaches for evaluation of bioactive compounds from Peru cocoa by-products for commercial applications

María de la Luz Cádiz-Gurrea [1,2*], Álvaro Fernández-Ochoa [1,2], Francisco Javier Leyva-Jiménez [2*], Noelia Guerrero-Muñoz [1], María del Carmen Villegas-Aguilar [1,2], Sandra Pimentel-Moral [1,2], Fernando Ramos-Escudero [3,4] and Antonio Segura-Carretero [1,2]

1 Department of Analytical Chemistry, Faculty of Sciences, University of Granada, Fuentenueva s/n, E-18071 Granada, Spain; mluzcadiz@ugr.es; alvaroferochoa@ugr.es; nogumu_95@hotmail.es; marivillegas@ugr.es; spimentel@ugr.es; ansegura@ugr.es
2 Functional Food Research and Development Center, Health Science Technological Park, Avenida del Conocimiento s/n, E-18016 Granada, Spain; jleyva@cidaf.es
3 Unidad de Investigación en Nutrición, Salud, Alimentos Funcionales y Nutraceúticos, Universidad San Ignacio de Loyola (UNUSAN-USIL), Calle Toulon 310, 15024 Lima, Perú; diomedes.fernando@gmail.com
4 Facultad de Ciencias de la Salud, Universidad San Ignacio de Loyola, Av. La Fontana 750, 15024 Lima, Perú.

* Correspondence: mluzcadiz@ugr.es; jleyva@cidaf.es

Received: date; Accepted: date; Published: date

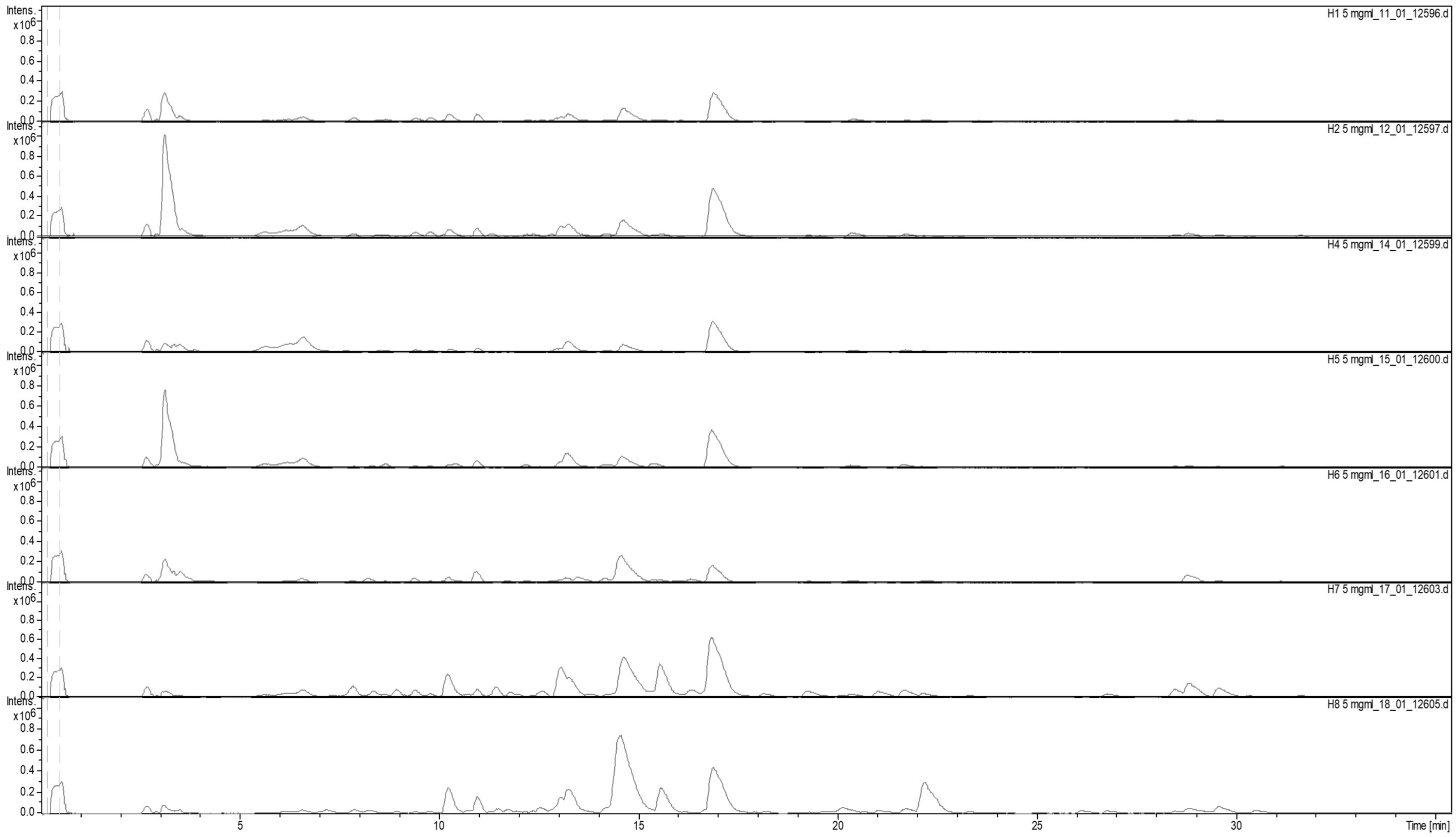

Figure S1. Base peak chromatogram of husk extracts.

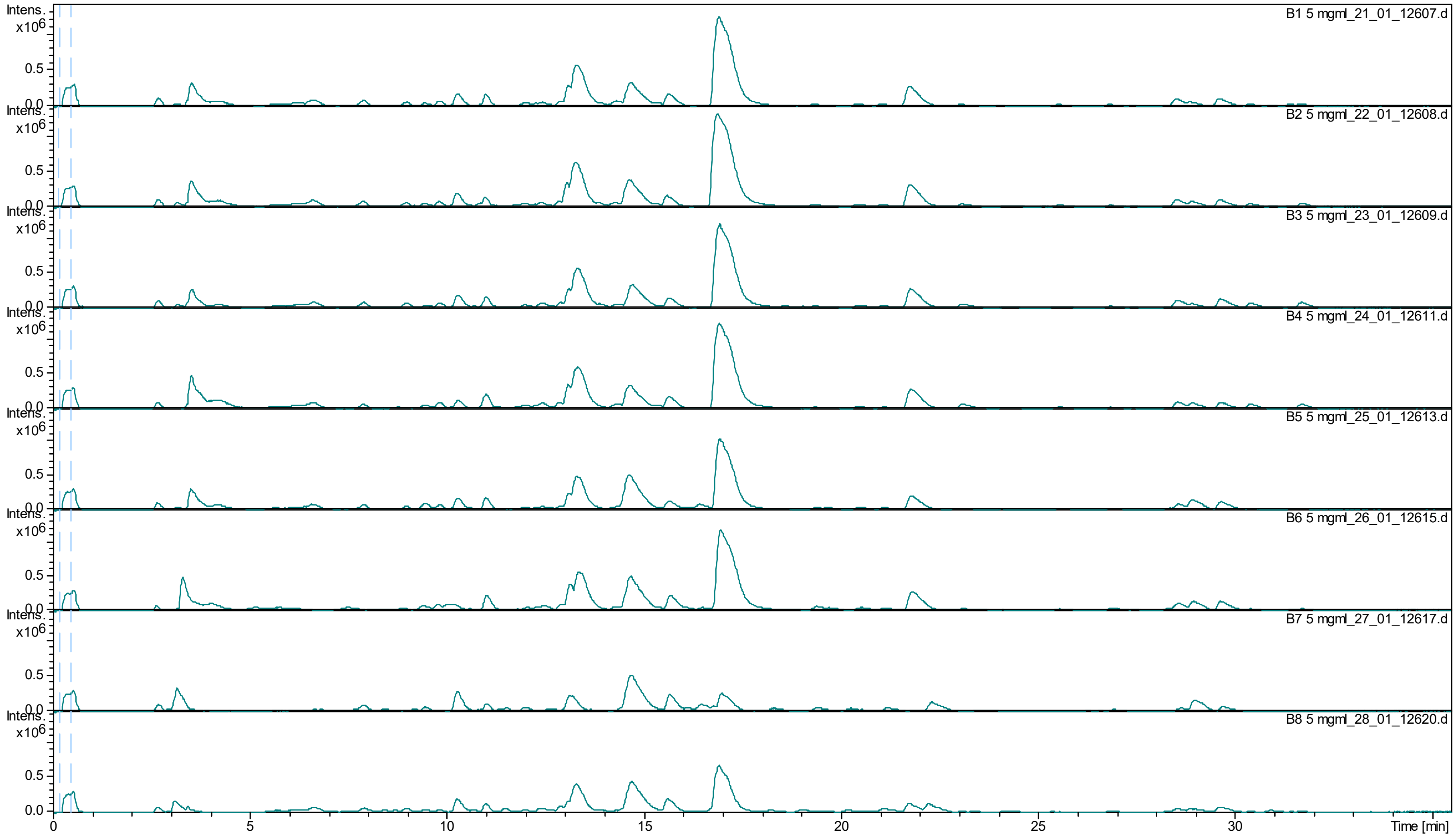

Figure S2. Base peak chromatogram of bean extracts.

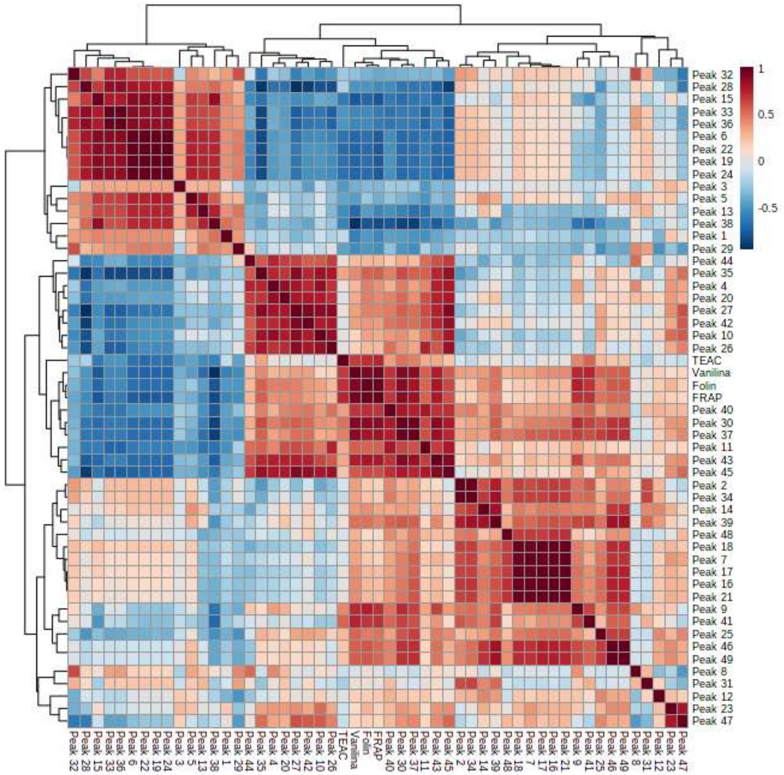

Figure S3. Correlation analysis using Pearson distance using all data obtained from husk and bean samples. (Intense red or blue colors show positive or negative correlations, respectively).

Table S1. Abundance of compounds from *Theobroma cacao* extracts by HPLC-ESI-TOF

Peak	RT (min)	H1	H2	H3	H4	H5	H6	H7	H8	B1	B2	B3	B4	B5	B6	B7	B8
**1**	3.1	4290925	15229827	16173660	1480737	11181725	3545189	1060563	597905	301611	651561	498555		417554	222763	4930343	2108712
**2**	3.5	1109835	1766259	1894916	2231009	2088824	3245786	139258	363045	5630942	6594567	3833622	8209820	5138648	7734950		
**3**	6.6	2234596	5836374	3952235	7862745	4670964	1604075	3658352	1607716	1799413	2221752	1868116	2115141	1578291		193168	1407512
**4**	7.9	683308	619224	1008852	270810	290811	369377	2126309	646581	1038002	928992	807082	836485	926819	1052583	1394693	788824
**5**	8.4	591988	463870	1045939	417470			1031602	92170								
**6**	8.6	473307	419156	599801	389985	654885	264532	94648	114202								
**7**	9.0	71537	176374	269435	32735	69194	128792	1297957	318204	563821	550750	563812	133515	428311	471629		439713
**8**	9.5	820386	982086	1365164	546795	377842	773480	1566960	339447	479324	575866	257721	734648	1216283	1045311	744158	448964
**9**	9.8	656641	754803	1209100	345974	280249	251069	579673	161785	752358	865802	770552	1009937	752280	823536	189827	335257
**10**	10.2	1324150	1401031	1819371	620448	471205	727653	3883675	3677583	2075439	2880987	2581855	1669822	2236452	1998171	4127118	2691281
**11**	10.9	965783	1015448	1624874	527451	861961	1403006	985607	1986686	1587194	1743509	1769551	2555552	2087596	2752025	1199746	1347036
**12**	11.3	99308	159163						160332		175534	115435	317716				
**13**	11.3	202847	479519	803316	148718	97879		6000091	382691							280809	
**14**	11.7	33279	53552	68611	52212	52082		116557	103531	161982	178037	114573	191485		147993		
**15**	11.7	119195	134477	216923	91507	122801	372297	148416	650758								42037
**16**	12.0	87869	127774	185605	178295	205245	43760	335939	271194	618000	694532	429780	840535	470240	518765		232602
**17**	12.4	254156	470449	788254	640986	146458	69352	352361	412946	738758	909776	687750	1184538	633670	987267		725436
**18**	12.8	492089	557760	1080169	737386	288638	156676	260051	756606	611009	706125	549811	909204	492272	931945		759882
**19**	12.9	675920	371767	396002	327505	199213	281683	58075	1161084								
**20**	13.0	971818	2029868	2655504	180336	1128823	535533	6165286	2886306	2591985	3198892	2188803	3177436	2141840	3861432	4307487	1222679
**21**	13.3	1548658	2503437	3990646	2222134	2774112	837236	4384341	4535736	13205342	14583516	12732746	14658464	10517797	12848014		8274238
**22**	13.4	333580	401989	565960	187968	450613	1137314	225426	854530								
**23**	13.9		107716	314977				695398	314540	555989	719058	613863					387615
**24**	14.1	727345	924340	1021955	635090	927628	1065871	160878	1370308								
**25**	14.3	208237	149771		174927			268007	105222	1176709	897165	660498	1020289	436977	593473		129681
**26**	14.6	3448379	4148478	7351788	1858124	2411954	6304727	10912777	21803953	8310332	10230985	8534128	8461537	13851283	13323489	14029637	11951075
**27**	15.6	464270	625465	1077279	333632	389283	551729	6258635	4186620	3174547	2959863	2542838	3185040	1994328	3569252	3916166	3289785
**28**	16.0	403109	261064	425749	109583	150026	305511										

Table S1. Cont.

**29**	16.3		154778	211911	83719	379844	1099612	2283598	285213					1298336	481040	1752872	
**30**	16.8	6618030	12447905	19451421	7294598	8632457	3507026	15898473	10646155	39602686	45452570	39513294	40048056	30102010	36768944	5727362	17706812
**31**	19.3	292301	424010	547598,72	213690	252913	203384	1638299	343890	313658	397543	414315	273485	430710	987971	859894	
**32**	19.5	223407	390308	508007,941	110974	162311	307053	598921						643493	732555		
**33**	20.3	733142	941424	1361361,22	538211	636139	549663	819309									
**34**	20.4	106109	163423	282614,834	105227	121432	64075	332957	224735	620903	628971	748219	553721	379379	835455		
**35**	21.0	88525	122570	232801,901	78348	108195	57457	397088	356620	621402	392879	555465	383959	549677	499771	893668	936451
**36**	21.3	245688	328438	507582,482	130851	117460	165168	293692									
**37**	21.6	402277	678720	1094421,76	574229	740209	195196	1579337	859464	6637529	7461859	6523091	6487568	4635660	6170161	160591	2331676
**38**	22.1	621705	421805	408400,398	451229	350797	591046	1045118	7427640							2924904	2540212
**39**	23.1	76790	301060	621279	116214	74202		338347	105174	494524	641172	1162884	1355546	376301	407167		
**40**	24.0			68739,674					128491	113109	345252	256412	278657	106283	54395		
**41**	25.5		474254	74491,739	56731	80338				517201	548335	458290	503187	229080	481697		
**42**	26.8	135028	119520	160054,733	92594	142644	139146	881400	654566	453338	415768	433889	355639	262194	762234	389266	243726
**43**	28.4	455077	528165	1165765,48	280128	402323	271593	2003532	582934	1694489	2047226	2136039	1828992	1481965	2195579	667061	839389
**44**	28.8	454462	843817	1271026,38	289247	482420	1674567	3616554	529700	947841	1711654	1176299	1620062	3096869	2783849	3528931	1207417
**45**	29.5	486918	528285	1213914	270639	369644	489334	2302394	1625290	1988492	2132785	2532437	1842377	2330070	2820670	1170178	1584921
**46**	30.3	68715	257127	505820	110429	51606		349532	92914	426661	752702	1046455	1113739	129274	340878		83066
**47**	30.9							78090	155367	115562	76112	177487					390040
**48**	31.3	120341		153579	55160	108155	154486	102937	194164	331531	103872	103888	192336	247054	231402		32056
**49**	31.6	88484	313953	625017	127768	53226		372322	65418	523339	837516	1340342	1066766	105905	301085		81067

RT, retention time; H, cocoa husk extracts; B, cocoa bean extracts

Table S2. ANOVA data for TPC, TFC and antioxidant assays of husk and bean extracts.

ASSAY	CONDITIONS		HUSK (sig.)	BEAN (sig.)	ASSAY	CONDITIONS		HUSK (sig.)	BEAN (sig.)	ASSAY	CONDITIONS		HUSK (sig.)	BEAN (sig.)	ASSAY	CONDITIONS		HUSK (sig.)	BEAN (sig.)
		2	,000	,033			2	,033	,000			2	,022	,000			2	,000	,860
		3	,000	,000			3	,000	,000			3	,000	,283			3	,000	,998
		4	1,000	1,000			4	,003	,000			4	,970	,000			4	1,000	,000
	1	5	1,000	,000		1	5	,000	,000		1	5	,773	,000		1	5	,142	,069
		6	,000	,006			6	,146	,000			6	,047	,001			6	,004	1,000
		7	,000	,000			7	,000	,000			7	,000	,000			7	,982	,000
		8	,000	,000			8	,000	,000			8	,000	,000			8	,000	,044
		1	,000	,033			1	,033	,000			1	,022	,000			1	,000	,860
		3	,000	,102			3	,000	,608			3	,000	,021			3	,003	,995
		4	,000	,022			4	,893	,000			4	,136	,029			4	,001	,003
	2	5	,000	,000		2	5	,008	,000		2	5	,328	,000		2	5	,113	,555
		6	,000	,000			6	,000	,000			6	,000	,999			6	,000	,746
TPC		7	,000	,000	TFC		7	,000	,000	FRAP		7	,000	,000	TEAC		7	,003	,000
		8	,000	,000			8	,000	,000			8	,000	,000			8	,023	,418
		1	,000	,000			1	,000	,000			1	,000	,283			1	,000	,998
		2	,000	,102			2	,000	,608			2	,000	,021			2	,003	,995
		4	,000	,000			4	,000	,000			4	,000	,000			4	,000	,001
	3	5	,000	,000		3	5	,000	,000		3	5	,000	,000		3	5	,000	,208
		6	,000	,000			6	,000	,000			6	,000	,061			6	,000	,985
		7	1,000	,000			7	,000	,000			7	,002	,000			7	,000	,000
		8	,000	,000			8	,069	,000			8	,204	,000			8	,947	,140
		1	1,000	1,000			1	,003	,000			1	,970	,000			1	1,000	,000
		2	,000	,022			2	,893	,000			2	,136	,029			2	,001	,003
	4	3	,000	,000		4	3	,000	,000		4	3	,000	,000		4	3	,000	,001
		5	1,000	,000			5	,093	,000			5	,999	,000			5	,183	,118
		6	,000	,009			6	,000	,001			6	,007	,010			6	,003	,000

		7	,000	,000			7	,000	,000			7	,000	,000			7	,994	,001
		8	,000	,000			8	,000	,000			8	,000	,000			8	,000	,177
	5	1	1,000	,000		5	1	,000	,000		5	1	,773	,000		5	1	,142	,069
		2	,000	,000			2	,008	,000			2	,328	,000			2	,113	,555
		3	,000	,000			3	,000	,000			3	,000	,000			3	,000	,208
		4	1,000	,000			4	,093	,000			4	,999	,000			4	,183	,118
		6	,000	,000			6	,000	,000			6	,002	,000			6	,000	,045
		7	,000	,000			7	,000	,000			7	,000	,000			7	,520	,000
		8	,000	1,000			8	,000	,000			8	,000	1,000			8	,000	1,000
	6	1	,000	,006		6	1	,146	,000		6	1	,047	,001		6	1	,004	1,000
		2	,000	,000			2	,000	,000			2	,000	,999			2	,000	,746
		3	,000	,000			3	,000	,000			3	,000	,061			3	,000	,985
		4	,000	,009			4	,000	,001			4	,007	,010			4	,003	,000
		5	,000	,000			5	,000	,000			5	,002	,000			5	,000	,045
		7	,000	,000			7	,000	,000			7	,000	,000			7	,001	,000
		8	,000	,000			8	,000	,000			8	,000	,000			8	,000	,028
	7	1	,000	,000		7	1	,000	,000		7	1	,000	,000		7	1	,982	,000
		2	,000	,000			2	,000	,000			2	,000	,000			2	,003	,000
		3	1,000	,000			3	,000	,000			3	,002	,000			3	,000	,000
		4	,000	,000			4	,000	,000			4	,000	,000			4	,994	,001
		5	,000	,000			5	,000	,000			5	,000	,000			5	,520	,000
		6	,000	,000			6	,000	,000			6	,000	,000			6	,001	,000
		8	,000	,000			8	,000	,000			8	,295	,000			8	,000	,000
	8	1	,000	,000		8	1	,000	,000		8	1	,000	,000		8	1	,000	,044
		2	,000	,000			2	,000	,000			2	,000	,000			2	,023	,418
		3	,000	,000			3	,069	,000			3	,204	,000			3	,947	,140
		4	,000	,000			4	,000	,000			4	,000	,000			4	,000	,177
		5	,000	1,000			5	,000	,000			5	,000	1,000			5	,000	1,000
		6	,000	,000			6	,000	,000			6	,000	,000			6	,000	,028

		7	,000	,000			7	,000	,000			7	,295	,000			7	,000	,000

Sig. = $p < 0.05$; TPC = Total Phenolic Content; TFC = Total Flavan-3-ol Content.
